# Supplementary material for: A study of Chinese enterprises’ business models to determine the impact of dynamic capabilities on innovation and performance
Source: PLoS One. 2025 Jan 27;20(1):e0310854. doi: 10.1371/journal.pone.0310854 (PMC11771891; doi:10.1371/journal.pone.0310854)
Supplement: S1 Questionnaire — (DOCX) [file pone.0310854.s001.docx]

**Questionnaire**

**A study of Chinese enterprises' business models to determine the impact of dynamic capabilities on innovation and performance**

Dear Sir/Madam,

I am a visiting scholar from Universiti Putra Malaysia (UPM). This research is part of my grant funding for the Soft Science Project of Ganzi Science and Technology Bureau in Sichuan Province (23kjjh0007 entitled “A study of Chinese enterprises' business models to determine the impact of dynamic capabilities on innovation and performance”. This is an academic exercise to gain much insight on factors that influence core technical competence towards competitive advantage in high-tech startups in China. This research is to offer findings and recommendations to assist the relevant stakeholders.

I would be grateful if you could spend 10–15 minutes to complete this survey. There are no right or wrong answers, and your responses to all the questions/statements should be entirely based on your own views and experiences. Please be assured that all the information you provide will be kept confidential.

For any inquiry please contact me at email: gs58620@student.upm.edu.my.

Your participation and support are very much appreciated. Thank you very much for your time.

Regards,

Dr. Yan. Jingwen

Universiti Putra Malaysia

**SECTION A:**

**DEMOGRAPHIC PROFILE**

| Instructions  This section intends to capture your demographic profile. Please tick (/) for your responses. Your response will remain anonymous. |
| --- |

1. Gender

| □Male | □ Female |
| --- | --- |

1. Your age

| □25-30 | □30-40 |
| --- | --- |
| □40-45 | □>45 |

1. Your position in company

| □CEO | □Vice-president |
| --- | --- |
| □Senior manager | □Manager |

1. How long you have worked in this company

| □>years | □3-5years |
| --- | --- |
| □5-10years | □>10years |

1. How many employees in the company

| □10-50 | □50-100 |
| --- | --- |
| □100-300 | □300-500 |
| 1. The annual sales revenue of your company | |
| □<5milion | □5-10milion |
| □10-15million | □15-30million |

1. Date of establishment

| □3years | □3-5years |
| --- | --- |
| □5-10years | □>10years |

1. Industry of the company

| □Information transmission | □Software and communication technology |
| --- | --- |
| □Artificial intelligence | □New energy and new materials |
| □Electronics and chips | □Chemical industry |
| □Consulting service industr | □Manufacturing industry |

1. Ownership of the company

| □Family firm | □Co. Ltd |
| --- | --- |
| □Social capital | □Sino-foreign jont venture |

**SECTION B**

**Enterprise Risk Management (ERM)**

| Instructions  This section focuses on your perception towards your PREFERRED enterprise risk management (ERM). Please read each statement below and indicate your response by circling the appropriate number (on a 7-point scale given below) which best describes your company. Please be reminded that there are no right or wrong answers. |
| --- |

Part B: Please rate (circle your response) the enterprise risk management (ERM) in your firm with regards to the following:

|  | Strongly Disagree | Disagree | Slightly Disagree | Neutral | Slightly Agree | Agree | Strongly Agree |
| --- | --- | --- | --- | --- | --- | --- | --- |
| 1.Our organization has implemented a comprehensive risk management policy to address significant risks that may impact our strategic objectives. | 1 | 2 | 3 | 4 | 5 | 6 | 7 |
| 2. A thorough analysis of risks and opportunities is the foundation for determining appropriate risk management strategies. | 1 | 2 | 3 | 4 | 5 | 6 | 7 |
| 3.We have well-defined procedures for implementing measures to mitigate identified risks. | 1 | 2 | 3 | 4 | 5 | 6 | 7 |
| 4.Top management and the board of directors are regularly informed of risks. | 1 | 2 | 3 | 4 | 5 | 6 | 7 |
| 5.The implementation of risk mitigation measures is closely monitored using standardized protocols. | 1 | 2 | 3 | 4 | 5 | 6 | 7 |

**SECTION C**

organizational agility (OA)

| Instructions  This section focuses on your perception towards your PREFERRED organizational agility (OA). Please read each statement below and indicate your response by circling the appropriate number (on a 7-point scale given below) which best describes your company. Please be reminded that there are no right or wrong answers. |
| --- |

Part C: Please rate (circle your response) the organizational agility (OA) in your firm with regards to the following:

|  | Strongly Disagree | Disagree | Slightly Disagree | Neutral | Slightly Agree | Agree | Strongly Agree |
| --- | --- | --- | --- | --- | --- | --- | --- |
| 1.We possess the agility to address customers' requirements promptly | 1 | 2 | 3 | 4 | 5 | 6 | 7 |
| 2. We demonstrate agility in swiftly adjusting our production/service delivery in response to demand fluctuations. | 1 | 2 | 3 | 4 | 5 | 6 | 7 |
| 3.We have the capability to resolve issues arising from suppliers promptly. | 1 | 2 | 3 | 4 | 5 | 6 | 7 |
| 4.They exhibit agility in implementing decisions to address market changes effectively. | 1 | 2 | 3 | 4 | 5 | 6 | 7 |
| 5.Innovation and reshaping our organization are active goals for our organization. | 1 | 2 | 3 | 4 | 5 | 6 | 7 |
| 6.In our view, market changes represent a favorable opportunity for rapid exploitation. | 1 | 2 | 3 | 4 | 5 | 6 | 7 |

**SECTION D**

Entrepreneurial orientation (EO)

| Instructions  This section focuses on your perception towards your PREFERRED entrepreneurial orientation (EO)**.** Please read each statement below and indicate your response by circling the appropriate number (on a 7-point scale given below) which best describes your company. Please be reminded that there are no right or wrong answers. |
| --- |

Part D: Please rate (circle your response) the entrepreneurial orientation (EO) in your firm with regards to the following:

|  | Strongly Disagree | Disagree | Slightly Disagree | Neutral | Slightly Agree | Agree | Strongly Agree |
| --- | --- | --- | --- | --- | --- | --- | --- |
| 1.My firm proactively takes action in response to competitors, prompting them to react accordingly. | 1 | 2 | 3 | 4 | 5 | 6 | 7 |
| 2.Innovating products, services, administrative techniques, and operating technologies is often in our wheelhouse, setting the pace for our competitors. | 1 | 2 | 3 | 4 | 5 | 6 | 7 |
| 3.Leaders in our organization strongly prefer introducing novel ideas and products before their competitors, consistently staying on top. | 1 | 2 | 3 | 4 | 5 | 6 | 7 |
| 4.Our company has successfully marketed a range of new products and services over the past five years. | 1 | 2 | 3 | 4 | 5 | 6 | 7 |
| 5.Creating a culture of innovation within the company is a top priority for our top managers. | 1 | 2 | 3 | 4 | 5 | 6 | 7 |
| 6.We consistently find our top managers capable of introducing new products and ideas ahead of the competition. | 1 | 2 | 3 | 4 | 5 | 6 | 7 |
| 7.High-risk projects with a substantial return potential are beautiful to our company. | 1 | 2 | 3 | 4 | 5 | 6 | 7 |

**SECTION E**

efficiency-centered BMI

| Instructions  This section focuses on your perception towards your PREFERRED efficiency-centered BMI. Please read each statement below and indicate your response by circling the appropriate number (on a 7-point scale given below) which best describes your company. Please be reminded that there are no right or wrong answers. |
| --- |

Part E: Please rate (circle your response) the efficiency-centered BMI in your firm with regards to the following:

|  | Strongly predictable | Predictable | Slightly predictable | Neutral | Slightly un predictable | Unpredictable | Strongly unpredictable |
| --- | --- | --- | --- | --- | --- | --- | --- |
| 1. We take active measures to reduce the price of our products or services. | 1 | 2 | 3 | 4 | 5 | 6 | 7 |
| 2. Our company takes active measures to reduce search and communication costs for our partners. | 1 | 2 | 3 | 4 | 5 | 6 | 7 |
| 3.The business model has a very low error rate in transaction execution. | 1 | 2 | 3 | 4 | 5 | 6 | 7 |
| 4.The Company takes active measures to speed up transactions. | 1 | 2 | 3 | 4 | 5 | 6 | 7 |
| 5.Transactions are transparent, and information and products and services can be tracked. | 1 | 2 | 3 | 4 | 5 | 6 | 7 |
| 6.Our company places importance on providing convenient and fast service to our customers. | 1 | 2 | 3 | 4 | 5 | 6 | 7 |
| 7.Overall, our business model offers high transaction efficiency. | 1 | 2 | 3 | 4 | 5 | 6 | 7 |

**SECTION F**

novelty-centered BMI

| Instructions  This section focuses on your perception towards your PREFERRED novelty-centered BMI. Please read each statement below and indicate your response by circling the appropriate number (on a 7-point scale given below) which best describes your company. Please be reminded that there are no right or wrong answers. |
| --- |

Part E: Please rate (circle your response) the novelty-centered BMI in your firm with regards to the following:

|  | Strongly predictable | Predictable | Slightly predictable | Neutral | Slightly un predictable | Unpredictable | Strongly unpredictable |
| --- | --- | --- | --- | --- | --- | --- | --- |
| 1. In general, the company's business model is novel. | 1 | 2 | 3 | 4 | 5 | 6 | 7 |
| 2.Our company is constantly introducing innovations in its business model. | 1 | 2 | 3 | 4 | 5 | 6 | 7 |
| 3.The extent to which the business model relies on trade secrets or copyrights. | 1 | 2 | 3 | 4 | 5 | 6 | 7 |
| 4.Our company has been awarded many patents due to the excellence of various aspects of its business model. | 1 | 2 | 3 | 4 | 5 | 6 | 7 |
| 5.This business model connects participants to transactions in a novel way. | 1 | 2 | 3 | 4 | 5 | 6 | 7 |
| 6. Business models offer an unprecedented variety and number of participants/goods/services. | 1 | 2 | 3 | 4 | 5 | 6 | 7 |
| 7.We are actively taking steps to combine products, information and services in new ways. | 1 | 2 | 3 | 4 | 5 | 6 | 7 |

**SECTION G**

SME performance

| Instructions  This section focuses on your perception towards your PREFERRED SMEs’ performance  . Please read each statement below and indicate your response by circling the appropriate number (on a 7-point scale given below) which best describes your company. Please be reminded that there are no right or wrong answers. |
| --- |

Part E: Please rate (circle your response) the SME performance in your firm with regards to the following:

|  | Strongly predictable | Predictable | Slightly predictable | Neutral | Slightly un predictable | Unpredictable | Strongly unpredictable |
| --- | --- | --- | --- | --- | --- | --- | --- |
| 1. Sales growth of the SME | 1 | 2 | 3 | 4 | 5 | 6 | 7 |
| 2. Profit growth of the SME | 1 | 2 | 3 | 4 | 5 | 6 | 7 |
| Market share for SMEs. | 1 | 2 | 3 | 4 | 5 | 6 | 7 |
| 4. Speed to market for SMEs. | 1 | 2 | 3 | 4 | 5 | 6 | 7 |
| 5. Market penetration rate (size) for SMEs | 1 | 2 | 3 | 4 | 5 | 6 | 7 |
| 6. The market value of SMEs. | 1 | 2 | 3 | 4 | 5 | 6 | 7 |
| 7. Net income of SMEs. | 1 | 2 | 3 | 4 | 5 | 6 | 7 |
| 8.Return on investment for SMEs. | 1 | 2 | 3 | 4 | 5 | 6 | 7 |

**END OF SURVEY**

**THANK YOU FOR YOUR PARTICIPATION!**
