# Supplementary material for: A study of Chinese enterprises’ business models to determine the impact of dynamic capabilities on innovation and performance
Source: PLoS One. 2025 Jan 27;20(1):e0310854. doi: 10.1371/journal.pone.0310854 (PMC11771891; doi:10.1371/journal.pone.0310854)
Supplement: Attachment — Submitted filename: Response to Reviewers.docx [file pone.0310854.s002.docx]

Dear Editors and Reviewers:

We would like to thank you for your valuable comments and helpful suggestions. We have studied these comments and suggestions carefully and made revisions to improve the quality of our manuscript. All revisions made to the manuscript were marked yellow. We hope the revised manuscript is satisfactory. Please let us know if you have concerns or questions about response to the referees.

**Journal Requirements**

1. Please ensure that your manuscript meets PLOS ONE's style requirements, including those for file naming. The PLOS ONE style templates can be found at https://journals.plos.org/plosone/s/file?id=wjVg/PLOSOne_formatting_sample_main_body.pdf and https://journals.plos.org/plosone/s/file?id=ba62/PLOSOne_formatting_sample_title_authors_affiliations.pdf

We sincerely thank you for your feedback, we have edited the format of the entire manuscript as well as the file naming.

2. Please ensure that you include a title page within your main document. We do appreciate that you have a title page document uploaded as a separate file, however, as per our author guidelines (http://journals.plos.org/plosone/s/submission-guidelines#loc-title-page) we do require this to be part

of the manuscript file itself and not uploaded separately. Could you therefore please include the title page into the beginning of your manuscript file itself, listing all authors and affiliations.

We sincerely thank you for your feedback, we have list the title page into the beginning of the manuscript file including all authors and affiliations.

3. We note that your Data Availability Statement is currently as follows: "All relevant data are within the manuscript and its Supporting Information files. "Please confirm at this time whether or not your submission contains all raw data required to replicate the results of your study. Authors must share the “minimal data set” for their submission.

We would like to thank you for your professional comment. The data has been uploaded as an attachment.

4. PLOS requires an ORCID iD for the corresponding author in Editorial Manager on papers submitted after December 6th, 2016. Please ensure that you have an ORCID iD and that it is validated in Editorial Manager.

Thank you for your comment, ORCID iD for the corresponding author has been added.

5. Please include your full ethics statement in the ‘Methods’ section of your manuscript file. In your statement, please include the full name of the IRB or ethics committee who approved or waived your study, as well as whether or not you obtained informed written or verbal consent. If consent was waived for your study, please include this information in your statement as well.

We added ethical considerations section in the line 585-594.

6. Please upload a new copy of Figure 1 as the detail is not clear. Please follow the link for more information:

Thank you for your comment, all Figures have been edited. Can see details in supporting information.

7. Please note that Table 4 is present, but Table 3 is missing.

Thank you for your comment, all Tables have been edited. Can see details in supporting information.

**Additional Editor Comments:**

Please carefully review our reviewers' comments as both suggested major revisions after carefully. Please carefully review our reviewers' comments as both suggested major revisions after carefully analyzing your manuscript. Although you have a very compelling subject, you are dealing with many concepts and variables and should be careful on your approach and readability of your article. Take special attention on the development of your framework and with details of your methodology

We sincerely thank you for your feedback. All reviewers' comments have been carefully revised and marked in the manuscript. Some inappropriate expressions have been rewritten, outdated references have been removed, and the literature review section has been polished. The methodology section carefully explains the sampling method, sampling frame, sampling population, and pilot test explain the reliability and validity of the questionnaire used. In the results section, the consistency and inconsistency between the research and previous studies are explained, and the reasons for such results are pointed out. In the practical application section, case study is added to better explain the suggestions and measures of this research.

**Reviewer1**

1.The manuscript needs a clearer explanation of how dynamic capabilities like enterprise risk management (ERM), organizational agility (OA), and entrepreneurial orientation (EO) directly impact SME performance. Detailed clarification of these mechanisms would strengthen the conceptual framework.

We sincerely thank you for your feedback which would help to improve the quality of our manuscript. We added these contents in the title of the manuscript: Relationship Between Enterprise Risk Management (ERM) and SMEs performance，Relationship Between organizational agility (OA) and SMEs performance，Relationship Between entrepreneurial orientation (EO) and SMEs performance.

2.More comprehensive methodological details are necessary, especially regarding the structural equation modeling (SEM) used. Information on model fit indices, assumptions testing, and justification for the sample size and sampling method should be included to ensure methodological rigor.

We sincerely thank you for your feedback**,** we added model fitting index, hypothesis testing, sample size, and sampling method in methodology part in the line of 595-616, Table1, Table 3and table 6.

3.The operational definitions and measurement scales for dynamic capabilities, business model innovation, and performance require thorough examination. The manuscript should specify how these constructs were measured and provide validity and reliability statistics for the scales used.

We sincerely thank you for your feedback. The manuscript has added operational definitions for each variable. explaining the basis for measurement, and conducting validity and reliability in pilot testing. Line 632-644,646-655,662-665,672-678.

4.The assertion that growth in efficiency BMI fully mediates the relationship between dynamic capabilities and SME performance needs robust statistical testing, possibly through bootstrapping techniques to provide confidence intervals for indirect effects.

We sincerely thank you for your feedback. The manuscript has added bootstrapping techniques to provide confidence intervals for indirect effects, Table 8 and line 756-762.

5. The discussion of results could be enhanced by exploring why certain dynamic capabilities might have stronger effects on business model innovation than others, and how this varies across different types of SMEs

We would like to thank you for your professional comment. We added those content in the main findings part, which can be seen on line 809-831.

6.Integration of literature needs improvement to build a compelling argument for the study’s contributions. The manuscript should discuss contradictory findings from previous studies to position the current research within the broader scholarly debate.

We would like to thank the reviewer for these constructive comments. We rewrite the introduction part to explain the contradictory findings and the research gaps. Line 31-124.

7.The practical implications section is too generic. A deeper discussion on how SME managers can leverage dynamic capabilities for business model innovation to enhance performance would be beneficial, potentially illustrated with examples or case studies.

We thank the reviewer for the positive and constructive comments regarding our paper. We added a case study to explain the practical implications, Line 834-895.

8.The limitations section should be expanded to address potential biases and the generalizability of the results. Future research directions could examine the role of external environmental factors such as market dynamics and technological changes.

Thank you for your comments. We added potential biases and the generalizability of the results, and market dynamics and technological changes in the limitations section, can be seen on line,897-904,911-933.

9.Clarify the usage of "Figure 1" for multiple diagrams to prevent confusion; each figure should be uniquely numbered.

Thank you for your comment. We have renumbered all tables and figures. Details on the supporting information part.

10.Standardize terminology such as "Efficiency BMI" and "Novelty BMI" throughout the document to avoid inconsistencies.

Thank you for your comment, we unified use Efficiency-cantered BMI and Novelty-cantered BMI.

11.Specify which version of AMOS was used for the analysis, as the manuscript mentions both AMOS 24.0 and AMOS 26.0, which could lead to credibility issues.

Thank you for your comment, we used AMOS 24.0, correct this clerical error.

12.Provide a justification or reference for the adequacy of the sample size for SEM to enhance the methodological section.

Thank you for your comment, We added G.power software for sample size in the line 603-608.

13.Improve the clarity in presenting hypothesis testing results, possibly through a structured table format, to distinctly communicate supported and unsupported hypotheses.

Thank you for your comment, we add a table 9 for hypothesis summary.

14.Update or remove outdated or irrelevant references to align the literature review with current research trends and improve the manuscript’s authority.

Thank you for your comment and I deeply apologize for the error in the literature review. I have carefully revised the literature and canceled outdated references.

15.Enhance the theoretical explanation for choosing specific mediators in the study to help readers understand the theoretical basis for expected influences.

We thank the reviewer for this insightful comment. We have rewritten the part, titled Mediating Role of BMI, line 481-457

16.Expand the ethical considerations section to include details about the rights informed to participants, such as confidentiality and withdrawal rights, to improve the study's ethical rigor.

We would like to thank the reviewer for this key comment. We added ethical considerations section in the line 585-594.

**Reviewer 2**

1.Regarding the color scheme consistency in Figure 1, it is essential that the colors assigned to 'resources,' 'strategy,' and 'performance' are uniformly aligned with their corresponding sections within the chart. Additionally, to more clearly depict the mediating effects, it is suggested that H6a and H6b be grouped together with H1a and H4, respectively. The same principle applies to H7a, H7b, H8a, and H8b.

We would like to thank the reviewer for this key comment, we changed the color and all H1-H5, H6a-H6f.can see **Fig1.**

2.Concerning the issue of sample selection, the article's choice of companies solely within Henan Province as the research sample may not be representative of the national standard. While the article justifies this by discussing the development of Henan Province, such reasoning may still lack justifies this by discussing the development of Henan Province, such reasoning may still lack persuasive power.

We would like to thank you for this valuable comment. We Added reasons for sample frame, please refer to line 564-582.

3.There is a numerical error; the correct figure should be 310. “The 132 questionnaires completed contained 132 valid responses.”

Thank you for your comment, we correct this clerical error.

4.This section of the content could be organized using a list format with numerical bullet points to improve clarity and readability. “The other items are：Our organization has implemented a comprehensive risk management policy to address significant risks that may impact our strategic objectives. A thorough analysis of risks and opportunities is the foundation for determining appropriate risk management strategies. We have well defined procedures for implementing measures to mitigate identified risks. Top management and the board of directors are regularly informed of risks. The implementation of risk mitigation measures is closely monitored using”

Thank you for your comment. We have numbered each variable's question item, line 655-661, line665-671, line 678-687, line 695-701.

5.There is a discrepancy between the article's conclusions and the empirical findings. The empirical results indicate that the direct effect of F5 on F6 is not significant, whereas the conclusion drawn in the text states that the direct effect of F5 on F6 is significant. This inconsistency also leads to issues in the analysis of the empirical results regarding the mediating effect. This issue is prevalent in multiple parts of the article, such as the description in the abstract: "Despite the insignificant indirect relationship between enterprise risk management (ERM), organizational agility (OA), entrepreneurial orientation (EO), novelty-centered BMI, and SME performance, this pathway is fully mediated by growth in efficiency BMI."

Please forgive our negligence, we carefully corrected the inconsistent parts in the text to ensure they are consistent with the research results. can see abstract as an example: line 10-18.

6.The description of the research content is vague. For instance, in the "main findings" section, it is mentioned that "Among the largely unanswered questions is how BMI affects ERM and firm performance." However, in your article, only the impact of ERM on BMI was tested, without addressing the influence of BMI on ERM. Such statements can cause confusion for the readers.

Thank you for your comment, we have rewritten the main finding part. see line769-831.

7.There is a mismatch between the research data and the conclusions. The data used in the article pertain to high-tech industrial enterprises and do not cover financial institutions such as banks. Therefore, the descriptions in the "practical implications" section of the article are inappropriate.

Thank you for your comment, we have rewritten practical implications, see line 834-895.

8.The quantification of the "firm performance" indicator is problematic. "Firm performance" is used as the dependent variable, measured by a composite of eight indicators, all of which are financial in nature and unrelated to the environment. Consequently, it is not possible to draw conclusions related to "corporate environmental performance."

Thank you for your comment; to avoid any misunderstandings we have corrected the main finding and practical implications sections

9.The basis for the classification of mediating variables is unclear. The article categorizes BMI into "efficiency-oriented" and "innovation-oriented" types, but it does not provide a clear explanation of how this classification is determined.

Thank you for your comment. We added explain the classification of BMI in the line 282-304.

10.Limitations and Future Research Directions. The article should provide a more detailed discussion of its research limitations, including the constraints of the data collection method, the limitations of the sample size, and other factors that may influence the results. It is recommended that the authors propose directions for future research, such as examining business model innovation in different cultural and economic contexts, or exploring how various types of dynamic capabilities affect the performance of SMEs across different industries.

Thank you for your comments. We added the limitations of data collection method, potential biases and the generalizability of the results, and market dynamics and technological changes in the limitations section, can be seen on line,897-904,911-933.

11.Data Accessibility. In accordance with PLOS ONE's policy, research data should be publicly accessible. The authors are requested to ensure that all relevant data are available in an appropriate public repository and to provide access information.

Thank you for your comments. The data of this manuscript has been uploaded in an attachment.

Sincerely,

Rahman

2024-6-5
