# Supplementary material for: A study of Chinese enterprises’ business models to determine the impact of dynamic capabilities on innovation and performance
Source: PLoS One. 2025 Jan 27;20(1):e0310854. doi: 10.1371/journal.pone.0310854 (PMC11771891; doi:10.1371/journal.pone.0310854)
Supplement: Attachment — Submitted filename: Response to Reviewers.docx [file pone.0310854.s003.docx]

Dear Editors and Reviewers:

We would like to thank you for your valuable comments and helpful suggestions. We have studied these comments and suggestions carefully and made revisions to improve the quality of our manuscript. All revisions made to the manuscript were marked yellow. We hope the revised manuscript is satisfactory. Please let us know if you have concerns or questions about response to the referees.

**Journal Requirements**

1. Please review your reference list to ensure that it is complete and correct. If you have cited papers that have been retracted, please include the rationale for doing so in the manuscript text, or remove these references and replace them with relevant current references. Any changes to the reference list should be mentioned in the rebuttal letter that accompanies your revised manuscript. If you need to cite a retracted article, indicate the article’s retracted status in the References list and also include a citation and full reference for the retraction notice.

We sincerely thank you for your feedback, we checked the references one by one in Google Scholar to ensure their accuracy.

**Additional Editor Comments**

1. Although you have had an approved decision, I strongly suggest you to

follow reviewer's 4 comments and some of reviewer's 5 suggestions as well to improve the quality of your manuscript before we find it ready for approval.

We sincerely thank you for your feedback, we accepted all the comments from Reviewer 4, and the specific response can be found in the following response to Reviewer 4. Regarding the issue of poor writing in Reviewer 5's comments, we have changed the structure of some sentences to ensure the readability for this manuscript.

**Reviewer 4**

1.Kindly replace many instances of the word 'cantered' with "centered"

We sincerely thank you for your feedback, we have corrected this typo.

2. I think this may be your most significant result, if possible highlight it a bit

more. EO -> Efficiency-centered BMI -> SME Performance, Indirect effect = 0.10,95% CI = [0.01, 0.17] excludes zero, Since the 95% confidence intervals exclude zero in all three cases, it indicates the mediating role of efficiency-centered BMI is statistically significant. So while efficiency-centered BMI did not have a strong direct effect on performance, the numerical evidence supports its significant indirect/mediating role in channeling the positive effects of ERM, OA and EO on SME performance in this study. Good result.

We sincerely thank you for your comments which would help to improve the quality of our manuscript, we have emphasized the most significant result in the line 804-811.

3. You also show that while novelty-centered BMI had a positive direct effect

on SME performance (path coefficient 0.125), it did not significantly mediate the relationships between ERM, OA, EO and SME performance based on the insignificant indirect effects. To me, this suggests that while novelty-centered innovations may have some benefits, they did not effectively translate the impacts of risk management, agility and entrepreneurial capabilities into better SME performance in this study's context.

We sincerely thank you for your feedback, indeed, although novelty- centered BMI may have some benefits, yet in the practical application scenarios of SMEs, they have not effectively transformed the impact of ERM, OA, and EO into better performance for SMEs. We have added some explanations that can be found in lines 783-790 of this manuscript.

4. I found two contradictions in the paper:

A. There is a contradiction between the positive direct effect of novelty-centered BMI on SME performance (path coefficient 0.125, p<0.001), and the lack of a mediating effect between novelty-centered BMI and the relationships of ERM, OA, EO with SME performance.

We sincerely thank you for your comments on the one hand, we deeply admire your meticulousness and professional, and on the other hand, we feel ashamed of our carelessness and lack of rigor. After careful verification by all editors, we have once again verified the data analysis results using by SPSS and AMOS. We found that the reason for these contradictions is that our data processing authors carelessly marked items ES1 and AI1-AI6 as F5(Efficiency-centered BMI) in Table 4, and mistakenly marked ES2, ES3, and CT1-CT5 as F4(Novelty-centered BMI), resulting in contradictory conclusions. F4 should be Efficiency-centered BMI, and F5should be Novelty-centered BMI. We have corrected this error in **Table 4** of the text.

There is a theoretical contradiction in using the dynamic capabilities view, which emphasizes continuous innovation and change, while the results favor efficiency-centered BMI over more novel, radical innovations.

We sincerely thank you for your comments, dynamic capabilities often seen as radical (novel) innovation. Teece (2018 pointed out dynamic capabilities are not limited to driving disruptive innovation (Teece, D. J. (2018). Business models and dynamic capabilities. Long Range Planning, 51(1), 40–49. https://doi.org/10.1016/j.lrp.2017.06.007.) Dynamic capabilities also include the ability to sense market and technological changes, seize opportunities, and reconfigure or transform resources to adapt to new environments. These capabilities may manifest differently in different firms and environments so that dynamic capabilities may drive efficiency gains, not just radical (novel) innovation. firms can build dynamic capabilities that are tailored to them. Moreover, efficiency-centered BMI can be seen as an innovation, especially in resource-constrained and competitive market environments. efficiency-centered BMI help firms improve their financial performance and market position in the short term, thereby building up capital and experience for future radical innovations.

This explanation can be found in line 824-833 of the text.

B. The hypotheses propose that both efficiency-centered and novelty-centered BMI will positively influence SME performance (H4 and H5). But the results only support H4 for efficiency-centered BMI, contradicting H5? Please elaborate.

We sincerely thank you for your feedback. As you can see, there are some literatures to prove that Novelty-centered BMI positive impact on performance. we also expect the same results when conducting this study. However, we found that Novelty-centered BMI did not have a direct relationship with performance in the application scenarios of SMEs. We could have deleted H5, but we suggest that although H5 is not supported, it can still be an important finding, and even serve as a warning for SMEs to be very cautious when conduct Novelty-centered BMI. This not only brings academic significance, but also practical significance in practical applications.

Moreover, we have added some possible reasons to explain this result, which can be found in line 783-790, and 804-811 of the text.

C. The literature review section highlights studies indicating ERM can uncover opportunities for BMI and innovation. However, the results show ERM only relates to efficiency-centered BMI, not novelty-centered BMI for SMEs.

We sincerely thank you for your feedback, when using AMOS for path analysis, only path F5 to F6 is not significant, indicating that Novelty-centered BMI has no direct impact on SMEs performance, yet ERM still have the positive impact on both Novelty-centered BMI and Efficiency-centered BMI according to findings on Table 7. This result can be considered that in SMEs, if Novelty-centered BMI is not used as a mediator but as a dependent variable, then ERM has a direct impact on Novelty-centered BMI. This also leaves space for future research to explore the antecedents of successful implementation of Novelty-centered BMI in SMEs. A possible explanation for this result is that ERM encourages companies to conduct BMI, but not all BMI application necessarily led to performance improvement. ERM tends to avoid risks, while Novelty-centered BMI tends to extensive and adventurous innovation. These two effects may offset each other, so there is no significance improvement in SMEs performance.

This explanation can be found in line 783-790 of the text.

The paper argues SMEs may have limited resources to experiment with BMI, yet also cites examples of large companies like Apple and Disney successfully implementing novelty-centered BMI.

Thank you for your feedback. Abernathy & Utterback （1978&1996）suggested the A-U model as an explanation for the technological evolution of enterprises in industries mature.(Utterback JM. Mastering the dynamics of innovation. Harvard Business School Press; 1996 Oct 7.) Thus, for SMEs, technological innovation follows certain rules, often by imitating successful examples of large companies, then introducing technology for technological transformation, and finally independent innovation. From the above rules, it can be seen that after a large company achieves success in Novelty-centered BMI, SMEs through imitation and technological transformation will eventually enter the stage of independent innovation, which is similar to that of large companies. Therefore, using the examples of Apple and Disney is to better explain the significant performance improvement brought about by Novelty-centered BMI. We also reasonably assume that the results of Novelty-centered BMI in SMEs are the same. But the results are inconsistent and can be seen as an academic finding.

The case study recommendation for the SME Daxin emphasizes efficiency BMI through modular manufacturing, contradicting the proposal for a novel "smart home" business model leveraging entrepreneurial orientation.

Thank you for your comments, according to iResearch "China's Smart Home Market 2023, by the end of 2022, the penetration rate of smart homes in urban households in China has reached over 40%. The technology of connecting and controlling multiple devices through a centralized platform is relatively mature. Therefore, it is not an adventurous innovation from scratch. Personalized smart homes can improve user comfort and satisfaction, and on the other hand, improve efficiency. From the research results, it can be seen that EO has a direct impact on SMEs performance. Currently, China has introduced many policies to support the development of technology-based SMEs. The construction of smart homes can not only receive government funding support, but also ensure that Daxin will not be eliminated in the huge smart home market. We rewrote a sentence to avoid misunderstandingsin line 917-918.

Please justify (briefly) these potential contradictions in the theoretical framing versus the empirical results favoring efficiency over novelty for SME BMI.

We sincerely thank you for your comments which would help to improve the quality of our manuscript, we added the explanation on line of 818-831.

5. Your data sources are complex, it may be difficult for researcher students to provide such data. At line # 657 or # 589, you can mention an early stage AI knowledge paper: "However, Agrrawal (Managerial Finance, 2009) shows that the utilization of automated web-harvesting algorithms can easily provide the researcher with zero-cost machine readable datasets for further analysis.

We sincerely thank you for your comments, we have added this reference in the line of 610-612.

6. A brief link between CSR and SME can be mentioned: Stoian, M. C., & Plakoyiannaki, E. (2023). Understanding international CSR in SMEs. Journal of Small Business Management.

We sincerely thank you for your comments, we have added this reference in the line of 117-118.

**Reviewer 5**

1. In short, I am sorry that I cannot be positive at this stage of the research project. Currently, the research is at best at the exploratory phase. This can be a part of a paper in the future. Unfortunately, it is poorly written and was very to grasp the essential points and arguments. Some of the most critical sentences made no sense at all due to either typos or poor command of the language. As of now, not much can be learned from this. There is significant major additional work for it to meet publication standards.

It was not clear if H1 to H5 are replications of other papers? Perhaps, then the authors can think about a cross-cultural nuance to this project and see how these results map against studies in western countries. It is more akin to a white paper for a regional economic development project.

Most of all, I beseech the authors to have it read by peers internationally and have it corrected by a professional editor before submitting elsewhere. My apologies for this somewhat unpropitious message.

Thank you for your comments, it is precisely because the writing skills are still insufficient that there is room for improvement. I hope the next work can satisfy you.

Similar hypotheses can be found in the literature for H1 to H5, as these are our understanding theories. Based on those, we can hypothesis BMI as a mediator and explore whether ERM, OA, EO affects SMEs performance through BMI. Exploring the essential factors that affect SMEs performance from H1 to H5. It is precisely the innovation of this article.

Of course, given your insightful opinion, we have made structural adjustments to some sentences to make them more readable.

Sincerely,

Rahman

2024-6-29
