# Supplementary material for: A study of Chinese enterprises’ business models to determine the impact of dynamic capabilities on innovation and performance
Source: PLoS One. 2025 Jan 27;20(1):e0310854. doi: 10.1371/journal.pone.0310854 (PMC11771891; doi:10.1371/journal.pone.0310854)
Supplement: Attachment — Submitted filename: PLOS-ONE-JUNE-2024-PONE-D-24-10141R2.docx [file pone.0310854.s004.docx]

July 16, 2024

Dr. Marcelo Dionisio, Editor, PLOS ONE

Dear Dr. Dionisio,

Please find attached my review of Manuscript **PONE-D-24-10141R2, “A study of the impact of dynamic capabilities on business model innovation and performance of SMEs based on Chinese enterprises”**

This can be accepted subject to minor revisions. Currently, it still needs some polish in writing, and there are lots of small stylistic lapses, but is understandable.

Thank you. I look forward to hearing your decision.

Sincerely,

Sundar

[P.V. (Sundar) Balakrishnan](http://www.uwb.edu/business/faculty/sbalakrishnan)

*Founding Faculty & Professor of Marketing,*

[*University Distinguished Teaching Award*](http://www.washington.edu/facultystaff/awards/2011-recipients/p.v.-sundar-balakrishnan-distinguished-teaching-award)

*<http://www.uwb.edu/business>*

Review of **Manuscript #: PONE-D-24-10141R2 “A study of the impact of dynamic capabilities on business model innovation and performance of SMEs based on Chinese enterprises”**

it is much better written. However, it is still hard to grasp the essential points and arguments.

1) Please spell out the Acronyms when you first use them at the least.

Ex: INTRODUCTION,

L 41-42. “BMI helps companies maintain their competitive advantage [9] by generating activities beyond product and process innovation [10].”.

L 73: “Studies indicates that ERM significantly impacts BMI by enhancing risk..”

At this point you have not said what BMI or ERM stand for. (Luckily it is in the Abstract). Consider adding a Table of all Acronyms.

2) L 150: “we will use a hypothesis and a model proposed in Section literature review”
   Do you have only ONE hypothesis? Please rewrite.
3) L 151: “Methodology explains how these data were collected and how they were analyzed”
   Rewrite Note, In the Methodology Section, we explain …
4) L 660: “Independent variable”. Should be plural form.
5) L 861: Table 9 Hypotheses summary. H5 is listed after H6. Not sure why.
6) ABSTRACT: L 27: “This paper is based on dynamic capacities theory”. I think you meant Dynamic CAPABILITIES THEORY – not CAPACITY?
7) L 776: FIG 2. This adds nothing. Use this space, for example, to provide the values if possible in something like Fig 1.

Great job. Once again, PLEASE, have it corrected by a professional editor before final acceptance.
