# Supplementary material for: A study of Chinese enterprises’ business models to determine the impact of dynamic capabilities on innovation and performance
Source: PLoS One. 2025 Jan 27;20(1):e0310854. doi: 10.1371/journal.pone.0310854 (PMC11771891; doi:10.1371/journal.pone.0310854)
Supplement: Attachment — Submitted filename: Response to Reviewers.docx [file pone.0310854.s005.docx]

Dear Editor and Reviewer:

We would like to thank you for your valuable comments and helpful suggestions. We have studied these comments and suggestions carefully and made revisions to improve the quality of our manuscript. All revisions made to the manuscript were marked BLUE. Please let us know if you have concerns or questions about response to the referees.

**Additional Editor Comments**

1. I kindly ask you to perform one more round of minor revision, with focus on writing, for which I strongly suggest you to perform a professional English review to improve the quality and readability of your manuscript.

We sincerely thank you for your feedback, we have performed SCRIBBR for proofreading, and certificate is attached.

**Reviewer**

1. Please spell out the Acronyms when you first use them at the least. Ex: INTRODUCTION, L 41-42. “BMI helps companies maintain their competitive advantage [9] by generating activities beyond product and process innovation [10].”. L 73: “Studies indicates that ERM significantly impacts BMI by enhancing risk.”At this point you have not said what BMI or ERM stand for. (Luckily it is in the Abstract). Consider adding a Table of all Acronyms.

We sincerely thank you for your feedback, in the introduction section, we added explanations when using abbreviations for the first time, in order to avoid additional tables due to space limitations.

2. L 150: “we will use a hypothesis and a model proposed in Section literature review”Do you have only ONE hypothesis? Please rewrite.

We sincerely thank you for your comment which would help to improve the quality of our manuscript, we have rewritten the whole sentence.

3. L 151: “Methodology explains how these data were collected and how they

were analyzed” Rewrite Note, In the Methodology Section, we explain……

We sincerely thank you for your feedback, we have rewritten the whole sentence.

4. L 660: “Independent variable”. Should be plural form.

We sincerely thank you for your comment. We changed to plural form.

5. L 861: Table 9 Hypotheses summary. H5 is listed after H6. Not sure why. We sincerely thank you for your comment, our initial idea was to put together

insignificant results, so H5 was listed after H6. To avoid confusion, we have changed to normal sorting.

6. ABSTRACT: L 27: “This paper is based on dynamic capacities theory”. I think you meant Dynamic CAPABILITIES THEORY – not CAPACITY?

We thank you for your meticulous reading, we have corrected the typo.

7. L 776: FIG 2. This adds nothing. Use this space, for example, to provide the values if possible in something like Fig 1.

Thank you for your comment, we have titled Fig2 as: Standardized estimates of independent variables, BMI and SMEs performance.

Other than that, we have performed SCRIBBR for proofreading. We sincerely apologize for typos and unprofessional expression used by non-native writers. We will continue to learn and improve. Thank you again for your rigorous and precisions review, which has benefited us greatly.

Sincerely,

Rahman

2024-8-29
